# Rac1 as a multifunctional therapeutic target to prevent and combat cancer metastasis

**DOI:** 10.18632/oncoscience.74

**Published:** 2014-08-21

**Authors:** Christoph R. Arnold, Alshaimaa Abdelmoez, Gudrun Thurner, Paul Debbage, Peter Lukas, Sergej Skvortsov, Ira-Ida Skvortsova

**Affiliations:** ^1^ Laboratory for Experimental and Translational Research on Radiation Oncology (EXTRO-Lab), Dept. of Therapeutic Radiology and Oncology, Innsbruck Medical University, Innsbruck, Austria; ^2^ Department of Anatomy, Histology and Embryology, Innsbruck Medical University, Innsbruck, Austria; ^3^ Assiut University, Dept. Pharmaceutical Organic Chemistry, Assiut, Egypt

**Keywords:** head and neck squamous cell carcinoma, metastasis, treatment resistance, carcinoma stem cells, Rac1

## Abstract

Metastatic progression of malignant tumors resistant to conventional therapeutic approaches is an ultimate challenge in clinical oncology. Despite the efforts of basic and clinical researchers, there is still no effective treatment schedule to prevent or combat metastatic spread of malignant tumors. This report presents recent findings that could help in the development of targeted therapeutics directed against the most aggressive and treatment-resistant carcinoma cells. It was demonstrated that HNSCC carcinoma cell lines with acquired treatment resistance possessed increased number of cells with carcinoma stem cell (CSC) properties. Furthermore, resistant cells were characterized by increased expression of Rac1, enhanced cell migration, and accelerated release of proangio- and vasculogenic factors (VEGF-A) and influence on endothelial cell (HMEC-1) migration. Inhibition of Rac1 signaling in the treatment-resistant carcinoma cells can interrupt metastatic process due to anoikis restoration and decrease of cell migration. It is also suggested that carcinoma cells with repressed survival capacities will be characterized by reduced release of proangiogenic factors, resulting in the decrease of endothelial cell migration. Therefore targeting of Rac1-related pathways may be considered as a promising therapeutic approach to prevent or combat metastatic lesions.

## INTRODUCTION

Metastasis is the final step in the progression of malignant tumors. Unfortunately, therapeutic options in the arsenal to treat metastatic lesions are very limited, hence clinical outcome is usually poor. Therefore, novel strategies and approaches to predict and combat metastatic spread in cancer patients are urgently needed. Novel therapeutic methods against metastatic diseases can be developed only if differences in molecular features in primary and secondary tumors have been elucidated. Despite similarly unfavourable clinical outcomes in all carcinoma patients with metastatic spread, certain malignant tumors demonstrate particularly poor clinical outcomes; head and neck squamous cell carcinoma (HNSCC) is one of these.

HNSCC is considered to be one of the most aggressive tumors. Despite the efforts of clinical and basic researchers, overall survival rates in HNSCC patients have not changed over the past 30 years [1-3]. Ineffective local and systemic treatment followed by loco-regional or distant metastatic spread of the tumor is considered to be the main cause of tumor-related mortality in HNSCC patients [4-6]. Thus, overall survival is 6-9 months for patients with local tumor relapses including regional metastatic lymph nodes, and 3-4 months for metastatic progressive HNSCC patients [6, 7]. It is generally believed that the main reason for metastatic progression after curative treatment is the survival of carcinoma cells possessing the following capacities: ability for self-renewal, resistance to chemo-radiotherapy, and inclination for metastatic spread. All these capacities belong specifically to carcinoma stem cells (CSCs) [8].

Since CSCs are refractory to conventional therapeutic approaches, it would be useful to establish a preclinical *in vitro* model of treatment resistance, in order to elucidate the intracellular molecular mechanisms of CSC insensitivity to anti-cancer therapy. Indeed, we have recently generated two radiation-resistant HNSCC cell lines (IRR cells): FaDu-IRR and SCC25-IRR, which survived after repeated exposure of the parental FaDu and SCC25 cells to ionizing radiation (10 Gy, 16 MV X-rays) in an Electa Precise Linear Accelerator (Elekta Oncology Systems, Crawley, UK). The cells received a total dose of 100 Gy. The newly obtained IRR cells retained their radiation resistance even after 3 years of passaging [3, 9]. Additionally, the IRR cells possess not only radiation resistance, but are also insensitive to cisplatin, docetaxel, and EGFR blockers [3, unpublished data for docetaxel and EGFR inhibitors]. These facts have a profound clinical impact: HNSCC relapsing after radiotherapy could also be resistant to chemo- and targeted therapeutics, hence the arsenal of agents available to treat HNSCC recurrences would be very restricted.

**Figure 1:**
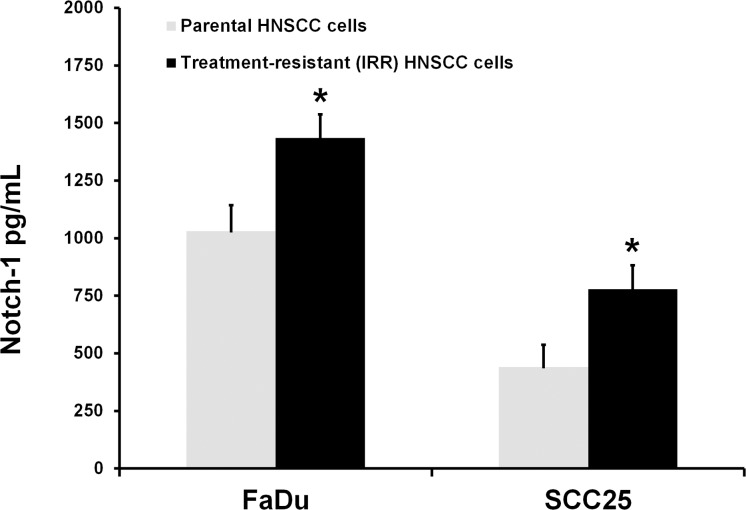
Notch-1 expression in HNSCC cell lines Notch-1 expression in the parental treatment-sensitive and treatment-resistant IRR HNSCC cells was determined by ELISA (Notch-1 ELISA Kit, RayBiotech, Inc, Norcross, GA, USA) as described in the manufacturer's instructions. *p < 0.05

It was next found that IRR cell lines were enriched in CD44**^high^**/CD24**^low^**/ALDH1+ carrying cells, which are also known as CSCs or tumor-initiating cells. Thus, FaDu- IRR and SCC25-IRR cell populations contained increased numbers of CSCs (18.65% + 5.26% and 13.06% + 3.98%, respectively). Treatment-resistant IRR HNSCC cells also showed enhancement of Notch-1 expression as evaluated using ELISA (Figure 1). The levels of Notch-1 expression fully corresponded to the levels of the increased number of CSCs in IRR cell lines. It is currently known that Notch-related signaling is markedly activated in CSCs and is involved in the regulation of CSC functions, such as enhanced clonogenic abilities and especially spheroid colony formation, and in the increased cell motility and aggressiveness of carcinoma cells, including altered processes of cell differentiation, proliferation and cell death [10-12]. Hence, our assumption that chemo- and radioresistant IRR cell populations contain higher number of CSCs has been proved.

Since IRR cells are enriched for CSCs, their protein signatures investigated by proteomic approaches could be re-considered to establish their involvement in intracellular and intratumoral processes, and possible molecular determinants characterising CSCs may be identified.

**Figure 2:**
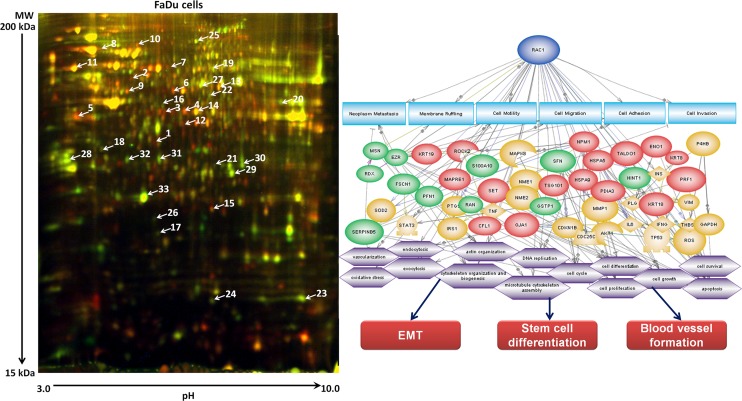
Protein patterns in radioresistant FaDu-IRR cells (A) Proteins differently expressed in parental and treatment-resistant FaDu cells were identified by two-dimensional differential gel electrophoresis (2-D DIGE) followed by MALDI-TOF/TOF mass spectrometry. (B) Identified proteins were evaluated for their common targets and activated processes in radioresistant HNSCC cells. Visual representation of molecular networks of DIGE- and software-found proteins and cell processes was performed using two versions of the software PathwayStudio 8.0 (Ariadne Genomics Inc.) [9] and PathwayStudio 10.3 (Elsevier B.V., Amsterdam, The Netherlands) based on valuable Medline citations from various investigators. Key for the represented shapes: ellipse – identified proteins; rectangles – cell processes. Green ellipses indicate DIGE-identified down-regulated proteins, red ellipses represent up-regulated proteins, orange ellipses indicate software-found proteins. Grey arrows show the relationship between cell processes and identified proteins [9].

**Figure 3:**
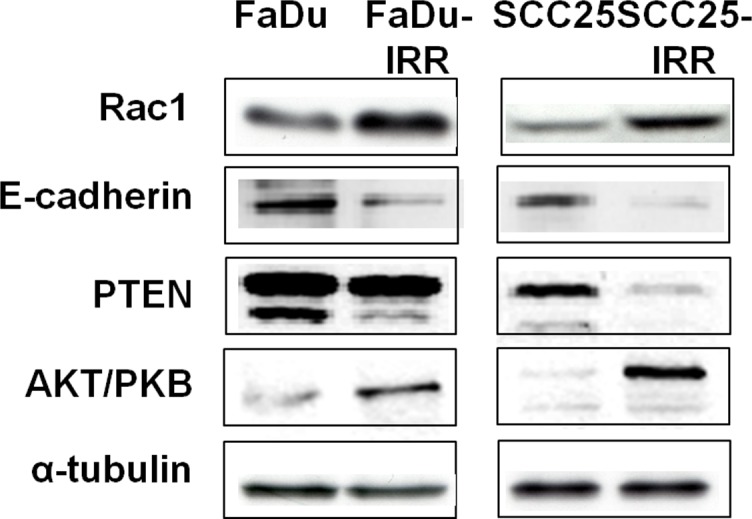
Modulation of expression of radiation-associated proteins in HNSCC cells Exponentially growing HNSCC cells were collected for Western blot analysis. Total protein extracts were prepared from the cells and then processed for immunoblotting using antibodies to detect Rac1, PTEN (Cell Signaling Technology, Beverly, MA, USA), E-cadherin, and Akt/PKB (Santa Cruz Biotechnology, Inc., Santa Cruz, CA, USA). α-tubulin was used as a loading control.

The software PathwayStudio 10.3 (Elsevier B.V., Amsterdam, The Netherlands) was used to analyze proteins showing differing expression in treatment-resistant IRR cells and in treatment-sensitive parental HNSCC cells, in order to determine their common targets (cell processes). In addition to our results reported in 2011 [9], which illustrated involvement of proteins in cell motility, migration, invasion, adhesion and neoplasm metastasis, further cell processes were also identified: epithelial-to-mesenchymal transition (EMT), stem cell differentiation, and blood vessel development (Figure 2). All the proteins identified using the proteomics approach had abundant relationships with Rac1 protein [3, 9]. Furthermore, Rac1 is closely linked to the intracellular pathways that are predicted to be activated in IRR HNSCC cells (Figure 3).

It is known that the mechanisms of metastasis include cell secession from the primary tumor, invasion into the surrounding tissue, intravasation into lymphatic or blood vessels, circulation and dissemination through the blood or lymphatic system, and extravasation from vessels followed by formation of the secondary tumors [13-15]. Thus, enhancement of cell motility, the formation of additional intratumoral vessels, and the activation of colony-forming abilities are critical for the metastatic spread of malignant tumors.

### Treatment-resistant HNSCC cells demonstrate resistance to anoikis

The first step in the metastatic process, cell secession from the primary tumor, usually triggers apoptosis (anoikis) [15, 16]. Therefore, if the detached carcinoma cell does not survive due to the initiation of anoikis, the process of metastatic spread will be interrupted already at the beginning. In contrast, if the detached carcinoma cell exhibits resistance to anoikis, the cell will survive and start its metastatic journey. Hence, the loss of sensitivity to anoikis significantly contributes to the enhancement of cancer cell metastasis.

Proteomics-based data have demonstrated that IRR cells have enhanced Rac1 expression as compared to parental HNSCC cells. Concomitantly, their expression of E-cadherin was down-regulated, resulting in decreased cell-cell junction formation and in the initiation of EMT in IRR cells. These events are reported to be specific for the initiation of metastatic spread in malignant tumors [17-19].

**Figure 4:**
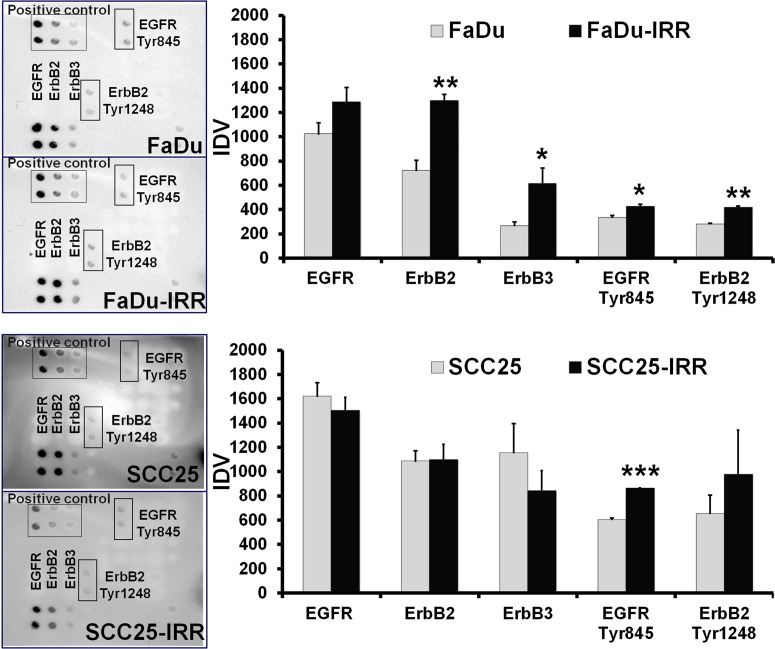
Receptor status (EGFR and ErbB2, ErbB3) in radio-resistant HNSCC cells Exponentially growing HNSCC carcinoma cells were analysed for expression of ErbB family members using RayBio® Human EGFR Phosphorylation Antibody Array 1 Kit (RayBiotech, Inc, Norcross, GA, USA). Integrated density values (IDVs) were normalized against the signal intensities of positive controls after background correction. Columns represent the mean value including standard deviation obtained from three independent experiments (*p < 0.05; **p < 0.01; ***p < 0.001).

Furthermore, treatment-resistant IRR HNSCC cells revealed activation of ErbB signaling (Figure 4). Thus, the FaDu-IRR cell line showed increased expression of total EGFR, ErbB2 and ErbB3 receptors, and both the IRR cell lines exhibited enhanced phosphorylation of EGFR (at Tyr845) and ErbB2 (at Tyr1248). FaDu-IRR and SCC25- IRR cells also showed amplified expression of Akt/PKB and down-regulation of phosphatase and tensin homolog (PTEN) (Figure 3). PTEN as a tumor suppressor acts as a repressor of the PI3K/Akt/mTOR pathway associated with activated ErbB family receptors. Loss or down-regulation of PTEN leads to activation of the pro-survival PI3K/Akt/ mTOR intracellular pathway followed by Rac1 activation, resulting in anoikis resistance [20-22]. Unfortunately it is still not clear how PTEN contributes to CSC formation, however it has been shown that activation of the Notch pathway results in down-regulation of PTEN in T-ALL cells [22, 23]. These data are in accordance with our own results presented in this report.

**Figure 5:**
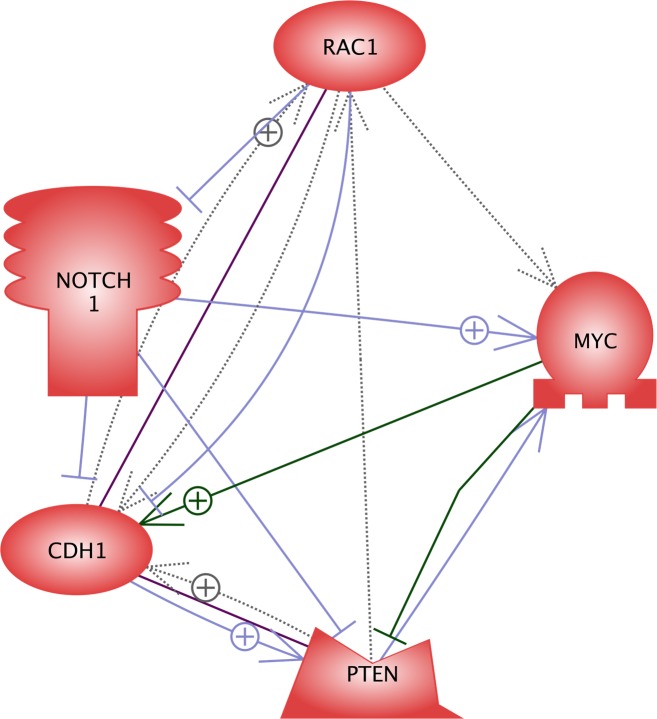
Direct relationships between proteins of interest Rac1, Notch-1, c-myc, E-cadherin (CDH1), and PTEN This network consisting of the analysed proteins was created using online software PathwayStudio 10.3 (Elsevier B.V., Amsterdam, The Netherlands) based on the Medline abstracts and full text publications.

Treatment-resistant IRR cell populations are characterized by increased numbers of cells which possess CSC markers (CD44**^high^**/CD24**^low^**/ALDH1+), have up-regulation of Notch-1 and repressed expression of PTEN. Additionally, Palomero et al. reported that concomitant overexpression of Notch-1 and c-myc reduced PTEN expression in T-ALL [22, 24]. It is interesting to note that up-regulation of the CSC specific marker CD44 variant 8-10 and c-myc indicates metastatically active and proliferating CSCs, whereas high expression of CD44 variant 8-10 accompanied by down-regulation of c-myc characterizes dormant CSCs [25, 26]. Recently we reported that the efficacy of combination treatment of HNSCC using ionizing radiation and the anti-EGFR antibody cetuximab depends on the expression of E-cadherin and c-myc [27]. Thus, E-cadherin repression accompanied by c-myc up-regulation upon therapy did not indicate improved tumor response to the combination of radiotherapy and cetuximab as compared to treatment with either ionizing radiation or cetuximab alone. Our research group is currently working to elucidate the relationships between Rac1, E-cadherin, c-myc and Notch-associated signalings in the treatment resistant HNSCC cells characterized by expression of CD44**^high^**/CD24**^low^**/ ALDH1+. Using the software PathwayStudio 10.3, we have found that Rac1, PTEN, E-cadherin, c-myc and Notch-1 have direct intracellular relationships (Figure 5). Thus, Rac1 can be suggested to be a central and main regulator of Notch-1, c-myc, E-cadherin and PTEN expression and activity [28-30]. It is possible to assume that cells detached from the primary tumors might survive due to the function of these activated pathways.

Since ErbB-related signaling is markedly up-regulated, it is logical to suggest that inhibition of ErbB pathways could be targeted to destroy carcinoma cells with migratory capacities. However, as we saw in our experiments, despite the activation of ErbB signaling in IRR cell lines enriched in CSCs, anti-ErbB agents (either blockers of EGFR (cetuximab, erlotinib) or both EGFR and ErbB2 (lapatinib)) were ineffective even to inhibit proliferation of treatment-resistant IRR cells. Hence, we hypothesize that inhibition of any of the other pathways implicated in the regulation of treatment resistance could be more effectively targeted to kill off highly aggressive carcinoma cells with metastatic properties. Since Rac1 was shown to be a central protein possessing the most abundant relationships with molecules and intracellular processes that are specifically altered in IRR cells [9], it is logical to suggest that Rac1 could play an essential role in preventing and combatting the metastatic spread of malignant tumors. Furthermore, it was also assumed that Rac1 can be considered as a marker of CSCs, because this molecule is involved in the maintenance of the CSC-specific characteristics, such as self-renewal, resistance to conventional therapeutic approaches, and enhanced cell migration and motility.

### Rac1 inhibition restores IRR cell sensitivity to ionizing radiation and chemotherapeutics

**Figure 6:**
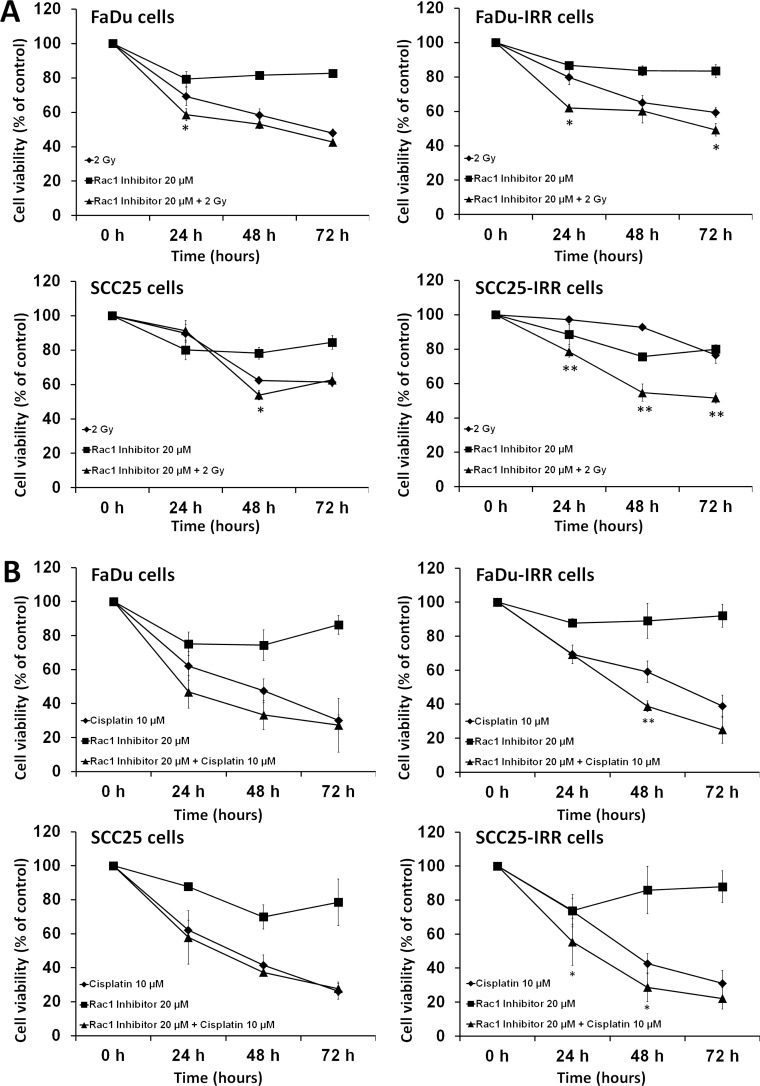
Rac1 inhibitor enhances the sensitivity of HNSCC to ionizing radiation or cisplatin (A) Parental FaDu, SCC25 and treatment-resistant IRR cells were exposed to ionizing radiation at a single dose of 2 Gy, to Rac1 inhibitor (20 μM) or to a combination of irradiation (2 Gy) and Rac1 inhibitor (20 μM). (B) parental and IRR HNSCC cells were treated with cisplatin at a clinically relevant single dose of 10 μM, with Rac1 inhibitor (20 μM) or with their combination, and then incubated for 72 hours. Cell viability and number of cells was evaluated using a Beckman Coulter Vi-CELL AS cell viability analyzer. Data are given as mean and standard deviation obtained from at least three independent experiments. *p<0.05, **p<0.01 - significance of differences in cell viability in HNSCC cells treated with ionizing radiation or cisplatin compared to their combinations wit Rac1 inhibitor [3].

As opposed to ErbB inhibitors, small molecule blockers of Rac1 successfully inhibited cell viability in HNSCC cells (Figure 6). Cell viabilities were markedly decreased when HNSCC cells were treated with ionizing radiation or cisplatin in combination with Rac1 inhibitor. Anti-proliferative effects of the combination treatment were more pronounced in therapy-resistant IRR cells than in parental HNSCC cells.

Table 1 shows how the doses of ionizing radiation and cisplatin required to reach 50% inhibition of clone formation in HNSCC were reduced in the presence of Rac1 inhibitor. These results are in accordance with recently published findings concerning the role of Rac1 in anchorage-independent proliferation and survival [31]. Therefore, it is possible to suggest that inhibiting Rac1 signaling in the detached carcinoma cells would combat anoikis resistance and thus result in cell death of the most treatment-resistant cells having increased metastatic activities, i.e. of the CSCs.

**Table 1:** Rac1-specific inhibitor enhances radiation- and cisplatin sensitivity in treatment-resistant HNSCC cell lines (IRR cells)

Cell line	Dose for radiation-induced 50% inhibitory effect on clone formation (IC50-IRRAD)(Gy)		Dose modifying factor (DMF) for IC50-IRRAD	Dose for cisplatin-induced 50% inhibitory effect on clone formation (IC50-CIS) (μM)		Dose modifying factor (DMF) for IC50-CIS
	Without Rac1 inhibitor	With Rac1 inhibitor (20 μM)		Without Rac1 inhibitor	With Rac1 inhibitor (20 μM)	
FaDu	2.96 ± 0.29	2.33 ± 0.01	***1.27***	2.82 ± 0.017	2.30 ± 0.06	***1.22***
FaDu-IRR	4.99 ± 0.53	2.83 ± 0.03	***1.76***	4.17 ± 0.094	2.11 ± 0.06	***1.97***
SCC25	3.49 ± 0.16	2.997 ± 0.11	***1.17***	2.33 ± 0.037	1.87 ± 0.04	***1.24***
SCC25-IRR	5.65 ± 0.44	2.34 ± 0.09	***2.42***	3.18 ± 0.056	2.25 ± 0.09	***1.41***

In order to evaluate the influence of Rac1 inhibitor on cell survival and clonogenic abilities of HNSCC cells after treatment with ionizing radiation or cisplatin, dose-modifying factor (DMF) was determined for radiation- or cisplatin-induced 50% inhibitory effect (IC50) without Rac1 inhibitor compared to IC50 when radiation or cisplatin were combined with Rac1 inhibitor. IC50 was determined using CalcuSyn software (Biosoft, Cambridge, United Kingdom) [3].

### Rac1 is a regulator of HNSCC cell motility

**Figure 7:**
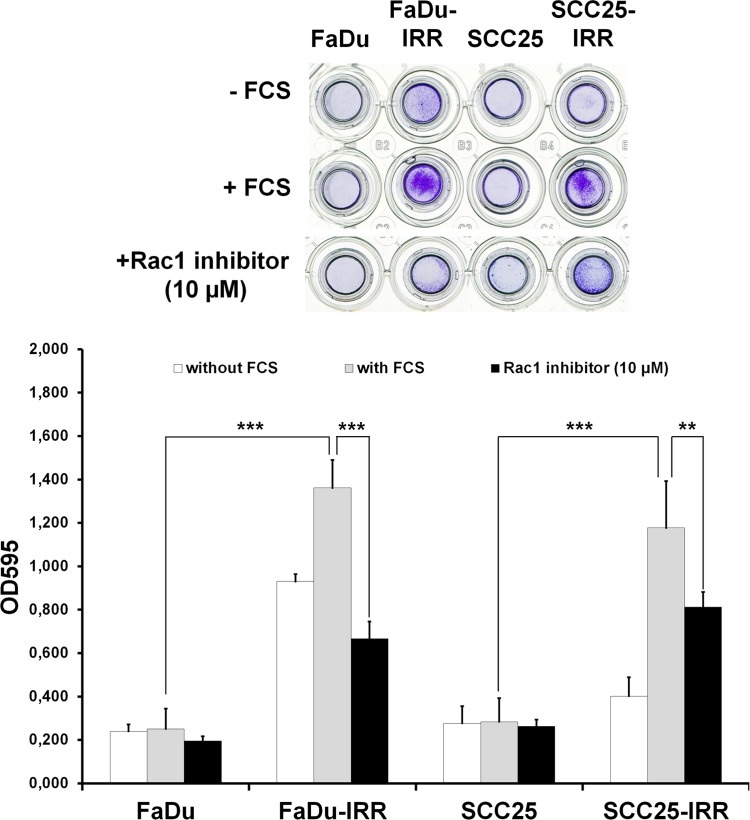
Effects of Rac1 inhibitor on HNSCC cell migration Differences in migration of parental and IRR HNSCC cells, and Rac1 inhibitor-induced repression of cell migration, were determined using a QCMTM 24-well colorimetric cell migration assay (Merck Millipore, Darmstadt, Germany), following the manufacturer's instructions. HNSCC cells harvested in the appropriate serum-free quenching medium were placed in the upper insert with an 8-μm pore size polycarbonate membrane. The lower chamber contained culture medium with chemoattractant (10% FCS). Plates were incubated for 24 hours at 37°C in a 5% CO2 humidified atmosphere. HNSCC cells that migrated through the membrane were stained and then subsequently extracted using extraction buffer. The optical densities of dye extracts were read at 560 nm using a microplate reader (Bio-Rad Microplate Reader 680, Bio-Rad Laboratories GmbH, Munich, Germany). **p < 0.01; ***p < 0.001 [9].

As shown in Figure 7, Rac1-overexpressing IRR cells demonstrated enhanced ability for cell migration. FaDu-IRR cells, characterized by more pronounced Rac1 expression, exhibited increased cell migration: by ~ 5.5-fold over parental FaDu cells, whereas in SCC25-IRR cells, which show lower Rac1 expression, migratory activity was slightly less enhanced: by ~4.2-fold. It is interesting to note that FaDu-IRR cells showed enhanced cell migration by ~4-fold even toward cell culture without chemoattractant (FCS). Hence, it is possible to assume that Rac1 is involved in regulation of the metastatic abilities of carcinoma cells. Indeed, Rac1 is described as a molecule playing a central role in cell motility, migration and invasion [31-34]. Since Rac1 is a critical regulator of metastatic activities of cancer cells, it is logical to suggest that inhibition of Rac1 expression or activity could result in the repression of metastatic potential of carcinoma cells. Indeed, Rac1 inhibitor (10 μM) blocked cell migration by ~ 2.0-fold in FaDu-IRR cells and by ~ 1.5-fold in SCC25-IRR cells (Figure 7). As seen from our results, Rac1 inhibitor demonstrated a more pronounced effect in decreasing cell migration in FaDu-IRR cells with their higher Rac1 expression. It seems that attenuation of Rac1 signaling in carcinoma cells can diminish their metastatic and invasive abilities.

### Rac1-overexpressing treatment-resistant IRR cells can enhance intratumoral angiogenesis

**Figure 8:**
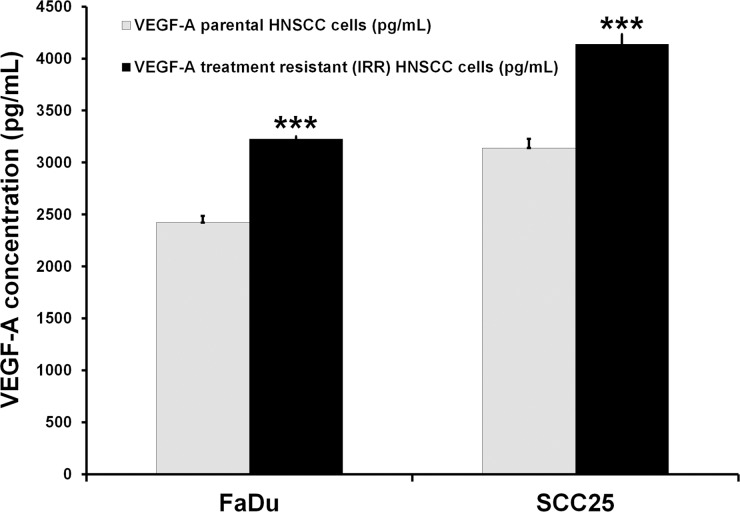
Assessment of VEGF-A concentrations in HNSCC cells VEGF-A concentrations were determined by ELISA (RayBiotech, Inc, Norcross, GA, USA) in the secretomes obtained from parental and treatment-resistant HNSCC cells. Briefly, HNSCC cells were seeded into the T75 flasks and incubated overnight. Then cells were washed and incubated in serum-free culture medium. Twenty-four hours later, serum-starved medium was collected for the evaluation of VEGF-A levels as described in the manufacturer's instructions. Results are shown as mean and standard deviation obtained from three independent experiments. ***p < 0.001.

**Figure 9:**
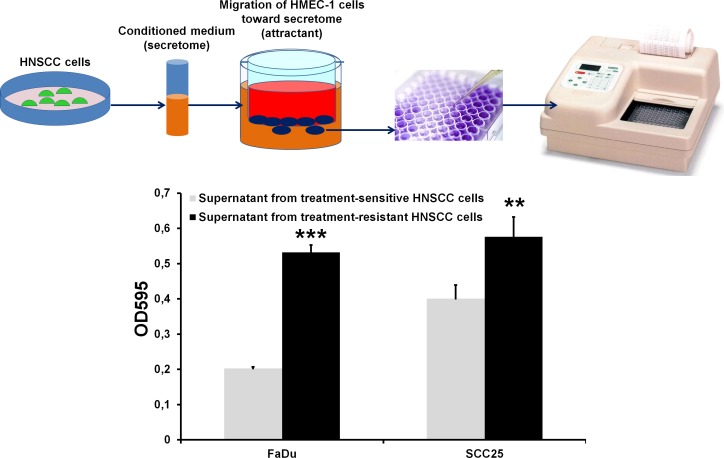
HMEC-1 cell migration toward secretomes collected from treatment-sensitive and treatment-resistant HNSCC cells Secretomes from parental and IRR HNSCC cells were collected as described in the legend for Figure 7. Migration of HMEC-1 cells was assessed using a QCM**^TM^** 24-well colorimetric cell migration assay (Merck Millipore, Darmstadt, Germany) as described in Figure 7. The lower chamber contained HNSCC secretomes as a chemoattractant. The optical densities of dye extracts obtained from the migrated HMEC-1 cells were read at 560 nm using a microplate reader. **p < 0.01; ***p < 0.001.

We next aimed to determine whether overexpression of Rac1 protein in treatment-resistant malignant tumors enriched in CSCs could contribute to metastatic spread via development of additional new blood vessels. Vascular endothelial growth factor (VEGF) and migration of endothelial cells are critical for vasculo- and angiogenesis, so we decided to measure VEGF-A release by treatment-resistant IRR cells and to evaluate changes they induced in the migration of human microendothelial cells (HMEC-1). Our preliminary data demonstrated that IRR HNSCC cells release more VEGF-A than do the treatment-sensitive parental HNSCC cells (Figure 8). Additionally, we used secretome obtained from parental and treatment-resistant HNSCC cells as a possible attractant for HMEC-1 cells. It was found that secretome from FaDu-IRR and SCC-IRR cells increased HMEC-1 migration by ~2.6- and ~1.4-fold compared to secretome obtained from treatment-sensitive parental FaDu and SCC25 cells, respectively (Figure 9). Administration of currently existing anti-angiogenic compounds (anti-VEGF or anti-VEGF-R antibodies or small molecules) does not markedly improve the therapeutic response of primary or metastatic lesions, because despite effective suppression of angiogenesis these compounds can increase the number of intratumoral CSCs [35]. Hence, we believe that inhibiting Rac1 signaling in HNSCC cells with CSC properties could also contribute to the decrease of neoangio- and vasculogenesis.

Unfortunately, the available data are very limited for further interpretation, because additional experiments using secretomes from Rac1-overexpressing cells and from HNSCC cells lacking expression of Rac1 have not yet been done. It is also planned to investigate VEGF-A secretion in subpopulations of CSCs and HNSCC cells with enhanced migratory activities, and to assess their influence on HMEC-1 migration. Additionally, *in vivo* experiments and clinical data are needed to confirm observed preclinical results.

## CONCLUSION

HNSCC cells surviving after ionizing radiation exposure possess diminished sensitivity to radiation treatment, chemo- and targeted therapeutics (EGFR blockers). Treatment-resistant cells enriched in CSCs also demonstrate increased expression of Rac1 accompanied by enhancement of cell motility that contributes to the processes of metastasis. IRR cell populations are characterized by higher numbers of CSCs, and release cytokines and growth factors (e.g. VEGF-A) activating migration of endothelial HMEC-1 cells. Inhibition of Rac1 expression and activity could either interrupt the metastatic process due to restoration of anoikis, or due to decrease of cell motility. It should therefore be possible to treat metastatic lesions by enhancing HNSCC cell sensitivity to ionizing radiation and chemotherapeutics. Furthermore, it is suggested that blocking Rac1-related pathways in treatment-resistant HNSCC cells enriched in CSC subpopulations could also diminish processes of neovascularization within malignant tumors.
